# The Brown Algae *Ishige sinicola* Extract Ameliorates Ovariectomy-Induced Bone Loss in Rats and Suppresses Osteoclastogenesis through Downregulation of NFATc1/c-Fos

**DOI:** 10.3390/nu14091683

**Published:** 2022-04-19

**Authors:** Mihyang Kim, Mihwa Park

**Affiliations:** Department of Food and Nutrition, College of Health and Welfare, Silla University, Busan 46958, Korea; mihkim@silla.ac.kr

**Keywords:** *Ishige sinicola*, postmenopausal osteoporosis, bone mass, osteoclastogenesis, bone resorption

## Abstract

Osteoporosis is characterized by reduction in bone mass and microarchitectural deterioration of the bone, which causes bone fragility and fracture susceptibility. *Ishige sinicola*, a brown alga, reportedly affects osteoblast differentiation. However, its protective effect on estrogen deficiency-induced bone loss has not been elucidated. This study aimed to investigate the effect of *I. sinicola* extract (ISE) on ovariectomy (OVX)-induced bone loss in vivo and osteoclastogenesis in vitro. Female Sprague-Dawley rats were randomly assigned to the sham-operated (SHAM) group and four OVX subgroups: SHAM, OVX, ISE20 (20 mg/kg), ISE200 (200 mg/kg), and estradiol (10 μg/kg). After 6 weeks of treatment, the bone mineral density (BMD), femur indices, and serum biomarker levels were measured. Furthermore, the effects of ISE on osteoclastogenesis and the expression of osteoclast-specific markers were measured. ISE administration improved the trabecular bone structure, bone biomechanical properties, BMD, and bone mineralization degree. In addition, the levels of serum bone turnover markers were decreased in the ISE group compared with those in the OVX group. Moreover, ISE inhibited osteoclast formation by downregulating NFATc1, TRAP, c-Src, c-Fos, and cathepsin K without any cytotoxic effects on RANKL-induced osteoclast formation. Therefore, we suggest that ISE has therapeutic potential in postmenopausal osteoporosis.

## 1. Introduction

Osteoporosis is a systemic skeletal disease characterized by impaired bone strength, diminished bone mineral density (BMD), microarchitectural degeneration, and increased fragility. Patients with osteoporosis are at a higher risk of bone fracture [1]. Osteoporosis is common in postmenopausal women and is linked to estrogen deficiency; bone loss affects 75% of postmenopausal women. Following menopause, the bone mass declines by 3–5% per year, and approximately 1.5 million women suffer from fractures caused by osteoporosis every year [2,3]. However, the pathogenesis of postmenopausal osteoporosis has not been completely elucidated. In addition, there are certain limitations to treating osteoporosis through drugs, and they may have adverse effects, such as endometrial cancer, breast carcinoma, and cardiovascular disease [4,5,6]. Therefore, it is essential to develop natural or synthetic substances with few negative side effects, replacing or minimizing the need for current drugs used.

Normal bone remodeling is accomplished by adjusting osteoblast-mediated bone formation and osteoclast-mediated bone resorption; however, in postmenopausal osteoporosis, the rate of bone resorption by osteoclasts is higher than the rate of bone formation by osteoblasts [7,8]. By providing a large mobilized pool of osteoclast progenitor cells, estrogen deficiency stimulates osteoclast formation [9]. Several cytokines control osteoclast differentiation, particularly a receptor activator of nuclear factor κB ligand (RANKL) and macrophage colony-stimulating element [10]. The binding of RANKL to the receptor activator of nuclear factor κB protein on the osteoclast precursor cell membrane activates the NF-κB pathway, which mediates osteoclast differentiation, survival, and activation [11]. In this process, RANKL prompts the expression of transcription factors, such as a nuclear factor of activated T-cells cytoplasmic 1 (NFATc1) and c-Fos [12]. NFATc1 is an important transcription factor that regulates many genes involved in osteoclastogenesis, including cathepsin K and tartrate-resistant acid phosphatase (TRAP) [13]. Hence, regulation of the osteoclastogenesis pathway is crucial for preventing and treating postmenopausal osteoporosis.

Ethnic peoples in eastern Asian countries have used crude marine algae extracts to treat diseases for centuries in folk medicine [14,15]. Historically, herbal medicinal marine algae (boiled in water and used as drugs in decoction) were first recorded in the medical literature about 2000 years ago in traditional Chinese medicine [16]. Marine algae abound in bioactive compounds with remarkable nutritional, pharmaceutical, and biomedical potential. Particularly, brown algae exhibit a variety of biological activities, such as anti-inflammatory, antioxidant, and anti-hyperlipidemia [17,18,19]. *Ishige sinicola*, a brown alga, is a popular food ingredient and marine herb in Korea and Japan (phylum Phaeophyta, class Phaeophyceae, order Ishigeales, family Ishigeaceae). *I. sinicola* was reported to have anti-inflammatory, antibacterial, and hair growth-promoting activities [20,21]. In addition, our previous study demonstrated that the *I. sinicola* extract (ISE) affects osteoblast differentiation via the bone morphogenetic protein 2/runt-related gene 2 signaling pathway [22]. However, its protective effects against bone loss induced by estrogen deficiency and osteoclastogenesis have not yet been reported.

Thus, in this study, we investigated the effects of ISE on postmenopausal osteoporosis using rats subjected to ovariectomy (OVX). Moreover, we evaluated the effect of ISE on osteoclasts and bone remodeling. This study might provide valuable insights regarding the therapeutic potential of ISE in postmenopausal osteoporosis.

## 2. Materials and Methods

### 2.1. Preparation of ISE

*Ishige sinicola* was collected (October 2018) from along the coast of Jeju Island, Korea. To remove the salt, epiphytes, and sand adhering to the surface, the sample was washed thrice with tap water, carefully rinsed with freshwater, and stored at −20 °C in a refrigerator. Thereafter, the frozen samples were lyophilized and homogenized with a grinder prior to extraction. ISE was prepared by extraction in 10 volumes of 80% methanol for 12 h at 37 °C thrice. Afterwards, the filtrate was evaporated at 40 °C to obtain the methanol extract. The ISE was thoroughly dried to completely remove solvent and stored in a deep freezer at −80 °C.

### 2.2. Animals and Treatment Regimen

Eight-week-old female Sprague-Dawley rats were purchased from ChemOn Inc. (Suwon, Korea). The rats were housed individually at a temperature of 23 ± 1 °C and a humidity level of 45 ± 5% in a 12 h/12 h light/dark cycle. In the experimental period, all rats were fed with basal 5L79 diets (PMI nutrition. LLC, Brentwood, MO, USA), and allowed free access to distilled water. All rats were treated strictly in accordance with the Silla University Institutional Animal Care and Use Committee’s guidelines for the care and use of laboratory animals. The animal study was reviewed and approved by the Animal Ethical Committee at Silla University, Busan, South Korea (approval no.: SUACAC-2018-008).

One week after acclimatization, rats were subjected to a bilateral sham or OVX surgery under ether anesthesia and maintained their diet for the experimental period. Three weeks after the operation, the OVX rats were randomly divided into the model (OVX, *n* = 7), low-dose ISE (ISE20, *n* = 7; 20 mg/kg body weight/day), high-dose ISE (ISE200, *n* = 7; 200 mg/kg body weight/day), and estradiol (E2, *n* = 7; 10 μg/kg body weight/day) oral administration groups. The dosage of ISE for rats in this study was determined based on the dosage utilized in Dietary Reference Intakes for Koreans (KDRIs) and calculated using the dose conversion table between humans and rats. The daily recommended single dose of seaweed is about 8 g/60 kg body weight, equal to 1.2 g of the ISE (yield 15%). This dose is equal to 20 mg of ISE/kg per day, considering an average adult body weight of 60 kg. The ISE-low dose group was administered a dose based on the weight ratio (20 mg/kg), and the ISE-high dose group was administered a ten times higher dose (200 mg/kg) according to the safety index. Rats in the ISE and E2 groups were intragastically administered a sample, which was dissolved in distilled water (in total volume 0.2 mL) via rat oral gavage needle (Natsume Seisakusho Co., Ltd., Tokyo, Japan). Rats in the SHAM and OVX groups were provided with equal volumes of distilled water (vehicle) as a control. In each group, the body weight of rats was monitored weekly. The rats were euthanized at the end of the experiment. Blood samples were obtained for isolation of serum, followed by biochemical analyses. The left/right femur and uterus were collected and weighed, and the tissue samples were fixed with 10% neutral formaldehyde prior to histological analysis. The fixed samples were stored at room temperature for later use.

### 2.3. Biochemical Parametric Analysis

The blood samples were centrifuged at 1300× *g* for 10 min at 4 °C after incubation at room temperature for 2 h. The serum was collected and stored at −20 °C for further biochemical analysis. N-terminal telopeptide of type 1 collagen (NTx) and β-C-terminal telopeptide of type 1 collagen (CTx) levels were determined using respective rat ELISA kits (Elabscience Inc., Houston, TX, USA). TRAP, RANKL, and osteocalcin (OC) levels were determined using respective rat ELISA kits (R&D Systems, Minneapolis, MN, USA). Serum alkaline phosphatase (ALP), Ca, and P levels were analyzed by the Dri-chem 500i automated clinical biochemical analyzer (Fujifilm Corporation, Tokyo, Japan).

### 2.4. Determination of the BMD and Biomechanical Properties

The complete left femur of the rats was collected, and the muscle and connective tissues were peeled off before analysis. The structural features of the trabecular bone of the proximal tibia and distal femur were determined by a high-resolution micro-computed tomography (μCT) scanner (VivaCT 80, Scanco, Zurich, Switzerland) according to the manufacturer’s instructions. The experimental conditions were as follows: voxel size, 18 μm (70 kvp, 114 μm, integration time = 200 ms); and FOV/diameter, 31.9 mm. Moreover, the biomechanical properties of the left femur were determined by the μCT analysis software (VivaCT 80, Scanco).

### 2.5. Tissue Histology

The left femur tissues were immediately collected and fixed with neutral formaldehyde for 72 h. Next, they were paraffin embedded according to the standard sampling and trimming procedures. The paraffin-embedded tissues were cut into 4-μm-thick sections and mounted on polylysine-coated microscope slides. For histological analysis, hematoxylin and eosin, and Safranin O staining (Sigma-Aldrich, St. Louis, MO, USA) were performed according to the manufacturers’ protocols (magnification: 40× and scale bar: 4 μm, respectively). Measurement of the BV/TV (%) was performed using the ImageJ software.

### 2.6. Cell Culture

RAW 264.7 murine macrophages were purchased from Korean Cell Line Bank (Seoul, Korea) and maintained in minimum essential medium-α (α-MEM; Gibco, Grand Island, NY, USA) supplemented with 10% fetal bovine serum (Gibco) and 1% penicillin/streptomycin at 37 °C in a humidified atmosphere containing 5% CO_2_.

### 2.7. Cell Viability Assay

Cell viability was evaluated using an MTT assay (Sigma-Aldrich). RAW 264.7 cells (5 × 10^3^ cells/well) were seeded in 96-well plates and treated with ISE (0, 10, 25, 50, and 100 μg/mL). MTT solution (0.5 mg/mL) was added to each well after 24 h, and the mixture was incubated for 4 h at 37 °C. The culture medium was then replaced with an equal volume of dimethyl sulfoxide to dissolve the formazan crystals. After incubation at room temperature for 30 min, the absorbance was measured at 540 nm using an ELISA microplate reader (Thermo Fisher Scientific, Waltham, MA, USA).

### 2.8. In Vitro Osteoclastogenesis Assay

RAW 264.7 cells (1 × 10^4^ cells/well) were cultured with or without RANKL (50 ng/mL) in the presence of ISE in 24-well plates. The medium was replaced every 2 days with fresh medium. The TRACP & ALP double-stain kit (Takara Bio Inc., Shiga, Japan) was used to identify osteoclasts, following the manufacturer’s instructions, following incubation of RAW 264.7 cells for 5 days. We considered TRAP positive multinucleated cells (MNCs) containing 3 or more nuclei as osteoclast-like cells and counted and captured them by a microscope. The cell culture medium was used to measure TRAP activity using a TRACP & ALP double-stain kit (Takara Bio Inc.) with a microplate reader at 405 nm.

### 2.9. Western Blotting

Cells were homogenized with an ice-cold lysis buffer containing 250 mM NaCl, 25 mM Tris-HCl (pH 7.5), 1% v/v NP-40, 1 mM dithiothreitol, 1 mM phenylmethylsulfonyl fluoride, and a protein inhibitor cocktail (10 μg/mL aprotinin and 1 μg/mL leupeptin). The cells were then centrifuged at 20,000× *g* for 15 min at 4 °C. The supernatants were used as the total protein extracts. The total protein contents were determined using the Bio-Rad protein kit (Bio-Rad Laboratories, Inc., Hercules, CA, USA) with bovine serum albumin as the standard. The lysates containing 30 μg of protein were subjected to electrophoresis on a 10% sodium dodecyl sulfate-polyacrylamide gel. Separated proteins were transferred electrophoretically onto a nitrocellulose membrane and blocked with 5% skimmed milk solution for 1 h. Next, NFATc1, c-Fos, β-actin (Cell signaling Technology, Beverly, MA, USA; Cat#4389, #2250, #3700), TRAP, s-Src, cathepsin K (Abcam, Cambridge, UK; Cat#ab191406, #133283, #187647) primary antibodies were added onto the membranes and then incubated overnight at 4 °C. After washing the membranes, they were incubated with the goat anti-rabbit or goat anti-mouse IgG horseradish peroxidase-conjugated secondary antibody (Cell Signaling Technology) for 1 h at room temperature. Each antigen antibody complex was visualized using the ECL Western Blotting Detection Reagents and detected by chemiluminescence with Luminescent Image Analyzer Davinch-Chemi™ (Davinch-K Co., Ltd., Seoul, Korea). Band densities of proteins were determined by an image analyzer (Multi Gauge V3.1; FUJIFILM Corporation, Valhalla, NY, USA) and normalized to that of β-actin.

### 2.10. Statistical Analyses

The data are represented as the mean ± SD of triplicate experiments. The statistical analysis was performed using SAS 9.3 software (SAS Institute Inc., Cary, NC, USA). The values were evaluated by one-way analysis of variance (ANOVA), followed by a post hoc Duncan’s multiple range test, and values of *p* < 0.05 were considered statistically significant.

## 3. Results

### 3.1. Effects of ISE on Body and Uterine Weight of Rats with OVX-Induced Osteoporosis

**Figure 1:**
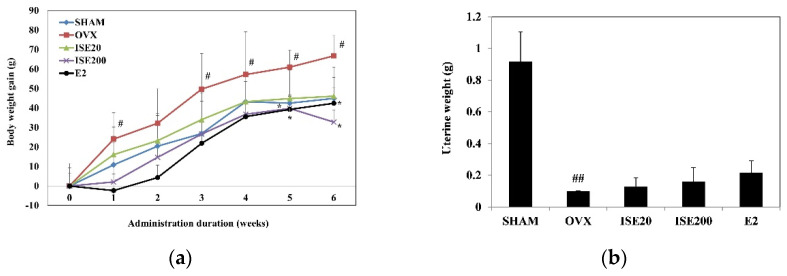
Effects of ISE on the (**a**) body and (**b**) uterine weight of ovariectomized rats. SHAM, sham-operated group; OVX, ovariectomy + vehicle; ISE20, OVX + low-dose ISE (20 mg/kg daily); ISE200, OVX + high-dose ISE (200 mg/kg daily); and E2, OVX + 17β-estradiol (10 μg/kg daily). Each value represents the mean ± standard deviation (*n* = 7). ^#^ *p* < 0.05 and ^##^ *p* < 0.01 vs. SHAM group; * *p* < 0.05 vs. OVX group. ISE: *Ishige sinicola* extract.

As shown in Figure 1a, the OVX group showed a critical increase in body weight gain compared to the SHAM group. The final body weight gain of rats in the OVX group (+66.78 g) was significantly higher than that of rats in the SHAM group (+44.94 g). However, the final body weight gain of rats in the OVX group was significantly reduced following the oral administration of 200 mg/kg of ISE (+32.8 g). These results indicate that ISE suppressed OVX-induced weight gain in ovariectomized rats.

To determine the effect of ISE on uterus weight in the OVX group, we administered ISE for 6 weeks and then weighed the uterus. As shown in Figure 1b, OVX caused significant atrophy of uterine tissue compared with the SHAM surgery, demonstrating the success of the surgical procedure (*p* < 0.05). The uterus weight of all rats in the OVX group was significantly decreased compared with that of rats in the SHAM group, owing to estrogen depletion. However, there were no significant changes in uterine weight in ISE20 and ISE200 groups compared to the OVX group.

### 3.2. Effects of ISE on the Serum Levels of Biochemical Parameters in Rats with OVX-Induced Osteoporosis

Serum levels of NTx, CTx, TRAP, RANKL, ALP, OC, Ca, and P were measured to assess the effect of ISE administration on biochemical markers in ovariectomized rats (Table 1). Serum NTx, CTx, TRAP, RANKL, ALP, and OC levels were significantly higher in the OVX group than in the SHAM group. Interestingly, the levels of these markers in the ISE groups were significantly lower than those in the OVX group. Compared with that in the SHAM group, the Ca level in the OVX group decreased significantly; however, ISE treatment tended to reverse the reduction in Ca level compared with the level in the OVX group. These results indicate that ISE administration in ovariectomized rats could moderate the OVX-induced increase in bone turnover rate and maintain Ca/P homeostasis in rats.

### 3.3. Effects of ISE on the Femoral BMD in Rats with OVX-Induced Osteoporosis

**Figure 2:**
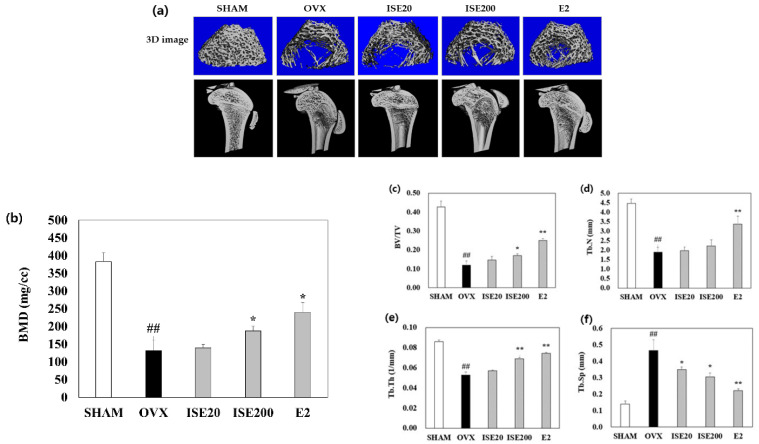
Effects of ISE on the trabecular bone morphometric parameters in ovariectomized rats. (**a**) Micro-computed tomography (μCT) images of the distal femur after 6 weeks of ISE administration. (**b**) Bone mineral density (BMD), (**c**) bone volume/tissue volume (BV/TV), (**d**) trabecular number (Tb.N), (**e**) trabecular thickness (Tb.Th), and (**f**) trabecular separation (Tb.Sp) as analyzed with μCT after 6 weeks of ISE administration. Each value represents the mean ± standard deviation (*n* = 7). SHAM, sham-operated group; OVX, ovariectomy + vehicle; ISE20, OVX + low-dose ISE (20 mg/kg daily); ISE200, OVX + high-dose ISE (200 mg/kg daily); and E2, OVX + 17β-estradiol (10 μg/kg daily). ^##^ *p* < 0.01 SHAM group vs. OVX group; * *p* < 0.05 and ** *p* < 0.01 OVX group vs. ISE20, ISE200, and E2 groups. ISE: *Ishige sinicola* extract.

The BMD and bone microarchitecture were analyzed using μCT to examine the effect of ISE on a rat model of OVX-induced osteoporosis. The structure of the trabecular bone of the distal femur and BMD of rats in the OVX group deteriorated compared with those in the SHAM group. However, treatment with high-dose ISE improved the trabecular bone structure and BMD (Figure 2a). Furthermore, compared with rats in the SHAM group, rats in the OVX group showed significantly decreased bone volume/tissue volume ratio, trabecular number, and trabecular thickness (Tb.Th). ISE administration, especially at the high-dose, enhanced the bone volume/tissue volume ratio, and Tb.Th in ovariectomized rats. In contrast, the trabecular bone separation in rats in the OVX group was higher than that in rats in the SHAM group. Treatment with ISE significantly reduced the trabecular bone separation in ovariectomized rats. These results demonstrate that treatment with ISE could prevent further bone loss in osteoporotic rats.

### 3.4. Effects of ISE on Morphological and Histomorphometrical Characteristics of Rats with OVX-Induced Osteoporosis

**Figure 3:**
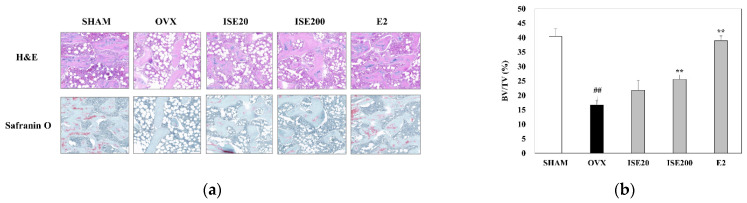
Effects of ISE on the trabecular bone tissue of the proximal femur in ovariectomized rats. (**a**) Histological examination of rat femoral heads tissue sections using hematoxylin and eosin (H&E) and Safranin O staining (magnification: 40× and scale bar: 4 μm, respectively). (**b**) Measurement of the BV/TV (%) using the ImageJ software. Each value represents the mean ± standard deviation (*n* = 7). SHAM, sham-operated group; OVX, ovariectomy + vehicle; ISE20, OVX + low-dose ISE (20 mg/kg daily); ISE200, OVX + high-dose ISE (200 mg/kg daily); and E2, OVX + 17β-estradiol (10 μg/kg daily). ^##^ *p* < 0.01 SHAM group vs. OVX group; ** *p* < 0.01 OVX group vs. ISE20, ISE200, and E2 groups. ISE: *Ishige sinicola* extract.

The femur of rats in all five groups was subjected to hematoxylin and eosin and Safranin O staining to observe histological changes. As shown in Figure 3a, the femoral trabeculae structure was sparse and the trabecular bone spacing increased in rats in the OVX group compared with that in the SHAM group. Deterioration of network connectivity and bone loss were clearly displayed at spongiosa zones of the femur in OVX group compared to those in the SHAM group, and it was diminished in the ISE-treated groups. The treatment of ovariectomized rats with ISE significantly increased the BV/TV (%) in a dose-dependent manner, compared to rats in the OVX group.

Similarly, Safranin O staining revealed the network connection deterioration, proteoglycan loss, and bone loss at the spongiosa zones at the femur of rats in the OVX group compared with that in rats in the SHAM group, and these effects were attenuated by ISE treatment (Figure 3b). These results suggest that ISE could ameliorate the estrogen deficiency-induced bone structural disorder in rats.

### 3.5. Effects of ISE on Osteoclast Differentiation

**Figure 4:**
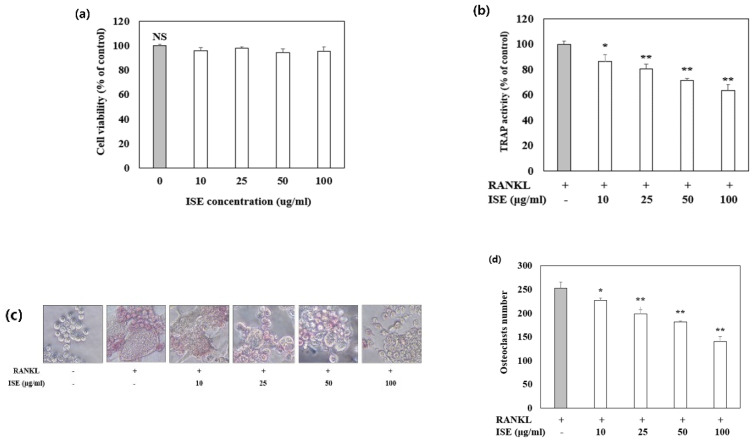
Effects of ISE on osteoclast differentiation in vitro. RAW 264.7 cells were cultured for 5 days with receptor activator of nuclear factor κB ligand (RANKL) (50 ng/mL) in the presence of various concentrations of ISE as indicated. (**a**) Cell viability was measured by MTT assay. (**b**) Tartrate-resistant acid phosphatase (TRAP) activity was assessed at an absorbance of 405 nm. (**c**) Cells were fixed and subjected to TRAP staining. (**d**) TRAP-positive cells were counted as osteoclasts. Each value represents the mean ± standard deviation (*n* = 3). * *p* < 0.05, vs. vehicle-treated control; ** *p* < 0.01, vs. vehicle-treated control; NS, not significant; ISE: *Ishige sinicola* extract.

A typical in vitro osteoclast differentiation model was used to study the effect of ISE on osteoclastogenesis. RANKL-treated RAW 264.7 cells were incubated with various concentrations of ISE and the osteoclast formation was assessed using TRAP staining. In response to RANKL stimulation, TRAP-positive multinucleated cells were formed, and ISE treatment significantly decreased the number of RANKL-induced osteoclasts without cytotoxicity (Figure 4). These findings indicate that ISE inhibited the differentiation of RANKL-induced osteoclasts in RAW 264.7 cells.

### 3.6. Effects of ISE on RANKL-Induced Expression of Osteoclastogenesis-Specific Proteins

**Figure 5:**
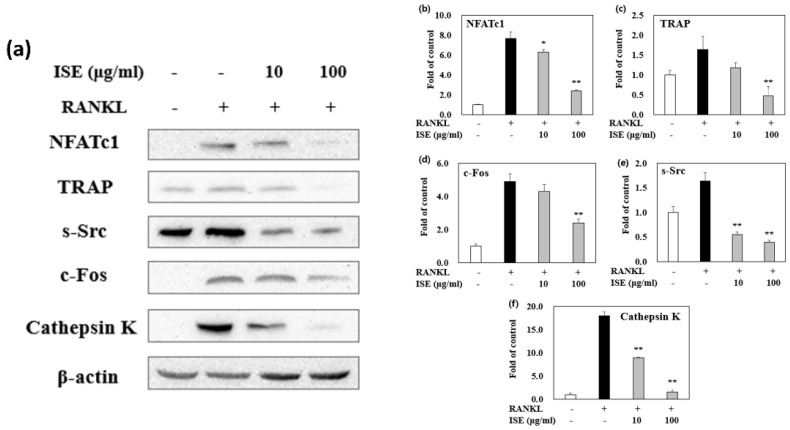
Effects of ISE on receptor activator of nuclear factor κB ligand (RANKL)-induced osteoclastogenesis-specific protein expressions. (**a**) The protein expression of the NFATc1/c-Fos signaling pathway-related markers, including the nuclear factor of activated T-cells cytoplasmic 1 (NFATc1), tartrate-resistant acid phosphatase (TRAP), c-Src, c-Fos, and cathepsin K, were detected by western blotting. (**b**–**f**) Relative expression was quantified by densitometry using Multi Gauge V3.1 and calculated according to the reference band densities of β-actin (mean, *n* = 3). ** *p* < 0.05, vs. vehicle-treated control; * *p* < 0.01, vs. vehicle-treated control; ISE: *Ishige sinicola* extract.

NFATc1 and c-Fos are crucial transcription factors for osteoclastogenesis. These transcription factors are produced in osteoclast precursors by RANKL stimulation, and their expression is automatically amplified. To examine the effect of ISE on osteoclast differentiation, we further evaluated the expression of RANKL-induced osteoclastogenesis-specific protein markers in the absence or presence of ISE (10 and 100 μg/mL). As shown in Figure 5, the protein expression levels of TRAP, c-Fos, c-Src, cathepsin K, and NFATc1 were significantly suppressed by ISE during osteoclast formation. These results strongly indicate that ISE may inhibit osteoclastogenesis.

## 4. Discussion

In the present study, we demonstrated that ISE inhibited OVX-induced bone loss in a rat model. Moreover, ISE acted as a potent inhibitor of osteoclast differentiation in RAW 264.7 cells by suppressing osteoclastogenesis via NFATc1/c-Fos downregulation.

OVX is a typical method of menopausal induction that lowers serum estrogen levels in rats, and it is commonly used in female osteoporosis studies [23]. Numerous studies have reported that a reduction in estrogen secretion leads to an increase in dietary intake, white adipose tissue, and, consequently, body weight, which is called metabolic obesity [24,25,26]. In addition, postmenopausal osteoporosis patients usually suffer from atrophy of some organs, especially the uterus and vagina [27]. Our results demonstrated that ISE administration could prevent OVX-induced weight gain. 

Bone turnover biomarkers reflect bone metabolic conversion, predict osteoporosis, and suggest fracture risk. Following menopause, the levels of biochemical markers of bone turnover, including those for bone formation and resorption, continue to increase with age [28]. Especially in osteoclastic resorption of the bone, CTx and NTx are cleaved and released into the bloodstream proportionately to the activity of bone resorption [29]. Osteoblasts and osteoclasts secrete ALP and TRAP, respectively; ALP is necessary for bone mineralization, whereas TRAP is associated with resorptive activity [30]. OC, a non-collagenous protein secreted by osteoblasts, reflects osteoblast activities, including bone turnover and osteogenesis [31]. Moreover, estrogen deficiency upregulates the production and activity of several cytokines, such as RANKL, which promote bone resorption, leading to increased levels of bone resorption markers [32]. In the present study, increased levels of bone formation biomarkers (ALP and OC) and bone resorption markers (CTx, NTx, TRAP, and RANKL) were observed in the OVX group, suggesting an increased bone turnover; however, the levels of these biomarkers were reduced in the ISE-treated groups. Further, Ca and P play phenotypic marker roles for bone formation [33]. In particular, Ca is one of the essential minerals that prevent osteoporosis. Established osteoporosis is generally connected with an imbalance of Ca due to problems such as the malabsorption of Ca and/or excessive Ca excretion [34]. In our study, the rats treated with ISE showed significantly increased serum Ca levels. Seaweed contains high amounts of magnesium and Ca in a form easily absorbed by the body. Collectively, these findings demonstrate that ISE prevented bone loss via the reduction in bone turnover and maintained Ca/P homeostasis in ovariectomized rats. 

Decreased bone density is one of the main elements influencing bone integrity, resulting in increased susceptibility to fractures and diminished bone strength [35]. Previous studies reported that OVX induces osteoporosis, leading to a significant deterioration in morphometric distal femoral parameters, such as the tissue volume, bone volume, Tb.Th, and trabecular number [36]. In this study, the oral treatment of ISE increased the tissue volume, bone volume, bone volume/tissue volume ratio, Tb.Th, and trabecular number in the tibia and femur of ovariectomized rats, while decreasing the trabecular separation. In addition, treatment of ovariectomized rats with ISE increased trabecular bone area relative to rats in the OVX group. Additionally, the findings of ISE treatment were consistent with those observed for E2 treatment, where the bone mass in both groups was maintained by inhibition of bone remodeling. Thus, ISE might exert protective effects on bone integrity.

The phytochemicals in marine algae extracts may offer promising novel treatment options for osteoporosis and other bone-related disorders. These alternative medicines may have better levels of activity and fewer side effects than the existing therapeutic agents. According to Jin et al., fucoidan, which is derived from brown algae, increases femoral density, and prevents microarchitectural deterioration in OVX rats [7]. In addition, extracts containing phlorotannins and phenolic compounds in brown algae have been shown to enhance osteoblast differentiation and subsequent mineralization [37]. Similarly, the extract of *Hizikia fusiforme*, a brown alga, has been reported to stimulate skeletal activity in zebrafish, ovariectomized mice, and calvarial mouse bones [38]. We previously suggested that ISE promotes bone formation through osteoblastic cells by stimulating osteoblast marker gene expression [22]. Consistent with these results, the present study demonstrated that ISE could be a beneficial therapeutic agent which prevents OVX-induced bone loss.

The abnormal bone resorption by osteoclasts is the major causal factor of osteoporosis. Thus, control of osteoclast differentiation might be a major therapeutic strategy for osteoporosis [10]. RANKL and macrophage colony-stimulating factors initiate osteoclastogenesis. They can bind to surface receptors of osteoclast precursors, a receptor activator of nuclear factor κB and cFms, respectively, and activate several major transcription factors [39]. NFATc1 and c-Fos are the two key target proteins involved in osteoclast formation after RANKL stimulation, whereas c-Fos is an important marker for NFATc1 activation [40,41]. NFATc1 regulates the expression of genes such as cathepsin K, c-Src, and TRAP, which are involved in osteoclast differentiation, fusion, and activation [42]. Interestingly, our study showed that ISE significantly inhibited osteoclast differentiation without cytotoxic effects in vitro. Furthermore, ISE suppressed the expression of osteoclastogenesis-related marker proteins, such as NFATc1, TRAP, c-Fos, c-Src, and cathepsin K. Consequently, ISE administration was able to directly suppress the expression of osteoclast-specific proteins. These results demonstrate that ISE inhibited the formation of osteoclasts by suppressing the expression of NFATc1/c-Fos and the RANKL-induced osteoclastogenesis pathway. However, future studies need to investigate the exact mechanisms of ISE or its bioactive compounds in inhibiting osteoclast formation. 

## 5. Conclusions

In conclusion, our findings demonstrated the efficacy of ISE to ameliorate the OVX-induced reduction in BMD and improve the loss of trabecular bone structure in ovariectomized rats. Furthermore, in vitro analyses suggested that the anti-osteoporotic effect of ISE might be mediated by its direct inhibition of osteoclastogenesis. ISE inhibited osteoclast differentiation by suppressing the induction of the NFATc1/c-Fos pathway in response to RANKL. Therefore, ISE could be a potential agent in preventing and treating OVX-induced osteoporosis.

## Figures and Tables

**Table 1:** Effects of *Ishige sinicola* extract (ISE) on serum biochemical markers in ovariectomized rats.

Variables	SHAM	OVX	ISE20	ISE200	E2
CTx (ng/mL)	1.02 ± 0.22	4.14 ± 0.19 ^##^	1.93 ± 0.21 **	1.16 ± 0.18 **	1.94 ± 0.09 **
NTx (ng/mL)	31.48 ± 3.29	79.40 ± 8.99 ^##^	35.92 ± 3.84 **	24.37 ± 2.98 **	33.42 ± 6.66 **
TRAP (U/L)	4.33 ± 0.42	16.58 ± 1.07 ^##^	9.55 ± 1.43 **	4.16 ± 0.09 **	5.94 ± 0.48 **
ALP (U/L)	180.14 ± 24.85	629.44 ± 16.30 ^##^	512.00 ± 21.21 **	223.00 ± 14.24 **	443.10 ± 18.4
Osteocalcin (pg/mL)	6.37 ± 0.28	10.21 ± 0.89 ^##^	6.38 ± 0.77 **	5.49 ± 1.06 **	4.77 ± 0.91 **
RANKL (pg/mL)	301.08 ± 3.23	423.38 ± 4.40 ^##^	351.85 ± 8.47 **	333.38 ± 9.02 **	341.46 ± 6.70 **
Ca (mg/dL)	13.23 ± 0.63	11.90 ± 0.29 ^#^	13.53 ± 0.84	13.78 ± 1.41 *	13.10 ± 0.26
P (mmol/L)	5.26 ± 0.42	6.67 ± 0.70 ^#^	6.20 ± 0.38	5.23 ± 0.84	5.21 ± 0.31 *

SHAM, sham-operated group; OVX, ovariectomy + vehicle; ISE20, OVX + low-dose ISE (20 mg/kg daily); ISE200, OVX + high-dose ISE (200 mg/kg daily); and E2, OVX + 17β-estradiol (10 μg/kg daily); CTx, β-C-terminal telopeptide of type 1 collagen; NTx, N-terminal telopeptide of type 1 collagen, TRAP, tartrate-resistant acid phosphatase; ALP, alkaline phosphatase; OC, osteocalcin; RANKL, receptor activator of nuclear factor κB ligand. Each value represents the mean ± standard deviation. (*n* = 7). ^#^ *p* < 0.05 and ^##^ *p* < 0.01 SHAM group vs. OVX group; * *p* < 0.05 and ** *p* < 0.01 OVX group vs. ISE20, ISE200 and E2 groups.

## Data Availability

Not applicable.
